# Milk fatty acid variability and association with polymorphisms in *SCD1* and *DGAT1* genes in White Fulani and Borgou cattle breeds

**DOI:** 10.1007/s11033-018-4331-4

**Published:** 2018-08-30

**Authors:** Isidore Houaga, Anne W. T. Muigai, Fredrick M. Ng’ang’a, Eveline M. Ibeagha-Awemu, Martina Kyallo, Issaka A. K. Youssao, Francesca Stomeo

**Affiliations:** 1 Department of Molecular Biology and Biotechnology, Pan African University Institute of Basic Sciences, Technology and Innovation, PAUSTI-JKUAT, P.O. Box 62000-200, Nairobi, Kenya; 2 Department of Animal Health and Production, Polytechnic School of Abomey-Calavi University, 01 P.O. Box 2009, Cotonou, Benin; 3 Department of Botany, Jomo Kenyatta University of Agriculture and Technology, P.O. Box 62000-200, Nairobi, Kenya; 4 Biosciences Eastern and Central Africa-International Livestock Research Institute (BecA-ILRI) Hub, P.O. Box 30709, Nairobi, Kenya; 5 Agriculture and Agri-Food Canada, Sherbrooke Research and Development Centre, 2000 Rue College, Sherbrooke, QC J1M 0C8 Canada

**Keywords:** Milk fatty acid variability, *SCD1*, *DGAT1*, Borgou, White Fulani, Benin

## Abstract

The stearoyl-CoA desaturase 1 (*SCD1*) A293V and acyl CoA: diacylglycerol acyltransferase 1 (*DGAT1*) K232A polymorphisms have been associated with significant variation in bovine milk fatty acid composition and unsaturation indices in western cattle breeds. This study aimed to estimate the milk fatty acid variability in indigenous Borgou and White Fulani cattle breeds of Benin, and the effects of the *SCD1* A293V and *DGAT1* K232A polymorphisms on milk and fatty acid composition and unsaturation indices. Thus, 85 Borgou and 96 White Fulani cows were genotyped for the *SCD1* A293V and *DGAT1* K232A polymorphisms and their milk and fatty acid composition and unsaturation indices were determined. Borgou presented milk with higher linoleic acid (P < 0.001), oleic acid (P < 0.05), C18 index (P < 0.001), total unsaturation index (P < 0.05), and lower total saturated fatty acid (SFA) compared to White Fulani. The *SCD1* VV genotype was associated with higher protein and lactose contents in White Fulani (P < 0.05). In Borgou, the *SCD1* AV genotype was associated with higher C14 and total unsaturation indices (P < 0.01), while the *SCD1* V allele was associated with decrease in C14 index (P < 0.05). In White Fulani, the *SCD1* VV genotype was associated with lower C18:1 *cis*-9 content (P < 0.05) while the *DGAT1* K allele was associated with increased total SFA (P < 0.05), and decreased C18 index (P < 0.05), total unsaturation index (P < 0.01) and total monounsaturated fatty acid (P < 0.01). The *SCD1* A293V and *DGAT1 K232A* may serve as genetic markers to improve milk fatty acid traits in Borgou and White Fulani breeds.

**Electronic supplementary material:**

The online version of this article (10.1007/s11033-018-4331-4) contains supplementary material, which is available to authorized users.

## Introduction

Milk and dairy products are important sources of energy, fat, high quality protein, vitamins, and minerals in human diets [1]. The fatty acids fraction of bovine milk fat is characterized by high amount (50–70%) of saturated fatty acids (SFA), 20–40% monounsaturated (MUFA) and low amount (1–5%) of polyunsaturated fatty acids (PUFA) [2]. Studies show that high quantities of dietary SFAs are associated with an increase in blood cholesterol and, therefore, with increased risk of atherosclerosis and coronary heart diseases in humans [3–5]. On the contrary, high concentrations of PUFAs in blood and tissue lipids is associated with positive benefits on cardiovascular diseases, mental health [6], exert anticancer effects [7] and play important role in preventing and/or treating various immunes disorders such as allergies [8, 9].

With regards to human health aspects, increasing unsaturated fatty acids in cow’s milk is an important selection goal [10]. The cow milk fatty acids are derived from two sources, the diet and the microbial activity in the rumen [11]. The fatty acids originating from the blood or from de novo fatty acid synthesis can be desaturated in the mammary gland [10]. Thus, the degree of unsaturation of milk fat is designated by a so-called unsaturation index, which is the ratio of the unsaturated product to the sum of the unsaturated product and the saturated substrate [10, 12]. Several studies showed that the variability of fatty acid composition in cow milk is affected by diet, breed, genetics, parity and stage of lactation [12–14]. In ruminant animals, milk fatty acid synthesis is regulated by several important enzymes including stearoyl CoA desaturase 1 (*SCD1*), acetyl CoA carboxylase, acyltransferases and fatty acid synthase [15]. Polymorphisms in diacylglycerol acyltransferase 1 (*DGAT1*) and *SCD1* genes coding for key enzymes in mammary lipid metabolism have been associated with significant proportion of variation in milk fatty acid composition and unsaturation indices in different cattle populations [10, 15–22].

On bovine chromosome 14, a lysine to alanine mutation in exon 8 (K232A) of *DGAT1* gene [23] has been shown to strongly affect milk composition and milk yield in different cattle populations [24–26]. Moreover, the *DGAT1* K232A polymorphism has been reported to be strongly associated with milk fatty acid composition in Dutch Holstein Friesian, Italian Brown, Holstein Crossbred and German Holstein dairy cattle breeds [10, 15, 19, 21, 27]. The *DGAT1* lysine variant (*DGAT1*K) was associated with higher saturated fat, a larger content of C16:0 and a small fraction of unsaturated C18 (C18:1 *cis* 9, C18:1 *cis* 11, C18:2 *cis* 9, 12, C18:3 *cis* 9, 12, 15, C18:1 *trans* 6, C18:1 *trans* 9 and C18:1 *trans* 11) and conjugated linoleic acid and C14:0 in Dutch Holstein Friesian [21]. The *DGAT1* A allele was associated with higher C18, conjugated linoleic acid (CLA) and total unsaturation indices and with lower C10, C12, C14 and C16 indices in Dutch Holstein Friesian cows [10].

The Stearoyl-CoA Desaturase 1 (*SCD1*) gene is mapped on chromosome 26 in cattle and expressed in a variety of tissues including adipose and mammary tissue [28]. Moreover, the *SCD1* enzyme contributes to the desaturation of SFAs into delta-9 unsaturated fatty acids [29]. A single nucleotide polymorphism SNP in exon 5 (C878T) of the *SCD1* gene leads to valine substitution by alanine at amino acid position 293 in the mature protein (Ala293Val). The *SCD1* A (Ala293) allele was associated with higher *cis*-9 C18:1 and total monounsaturated content as well as C14:1/C14 ratio in Italian Holstein [22]. However, data on the genetic variability of milk fatty acid composition and unsaturation indices are scarce in African indigenous cattle breeds and the few studies on *DGAT1* K232A polymorphism in African indigenous cattle did not show association with milk fatty acid composition [30–32]. In the study of Rahmatalla et al. [31], the *DGAT1* K232A genotypes showed significant effects on fat content in Sudanese indigenous Kenana and Butana cattle breeds. The study of Houaga et al. [32] mentioned that the *DGAT1* KK genotype was significantly associated with higher fat yield in White Fulani (P < 0.05). However, they did not investigate the milk fatty acid composition.

In Benin, indigenous White Fulani and Borgou cattle breeds are the main milk producers [32]. However, to date, there has been no study on *SCD1* A293V and *DGAT1* K232A influence on milk and fatty acid composition and unsaturation indices. Moreover, no detailed data on milk fatty acid composition of these breeds is available. Such data would be useful for gathering knowledge on nutritional value of indigenous cow milk for the dairy industry in Benin and for the opportunity to improve cow’s milk fatty acid composition with regards to human health. Therefore, the present study aimed to estimate the milk fatty acid variation in white Fulani and Borgou cattle breeds and the effects of the *DGAT1* K232A and *SCD1* A293V polymorphisms on milk and fatty acid composition and unsaturation indices.

## Materials and methods

### Sampling

**Fig. 1:**
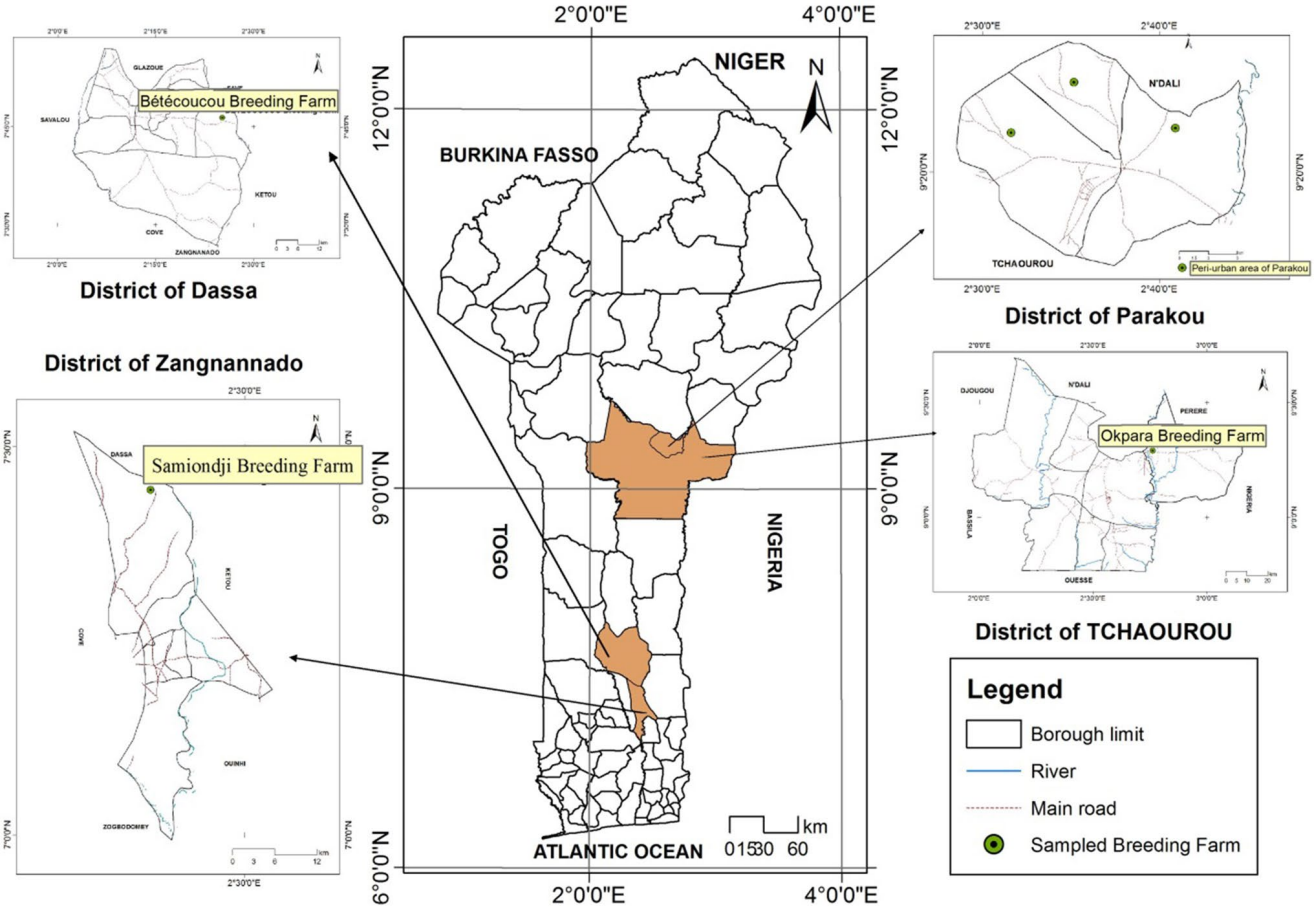
Map of Benin indicating the sampling sites

A total of 85 Borgou and 96 White Fulani indigenous cows were sampled from state owned farms (Betecoucou, Okpara and Samiondji) and privately owned farms in Benin between May–July 2016 (raining season). Blood and milk samples were obtained from three to ten cows per herd from a total of 17 herds. Only cows in lactation were randomly selected and sampled. The map of Benin indicating the sampling sites is presented in Fig. 1. The feeding system was based solely on natural grazing without concentrate supplementation. The cows were milked once a day in the morning. Additional meta data on sampled cows obtained from the livestock keepers and herders following a written consent permitting sampling included: age, lactation stage and parity number. Blood samples were collected from the jugular vein into 10 ml EDTA vacutainer tubes and immediately transported to the laboratory in a cool box containing ice and stored at − 20 °C until further analysis. Milk samples were aseptically collected into 50 ml falcon tubes each containing one tablet of Bonopol milk preservative (Systems Plus, Canada) and sent to Valacta laboratories (Valacta Laboratories Inc., Canada, http://www.valacta.com) for the analysis of milk components.

### Milk component analysis

Test-day milk fat percent, protein percent, milk urea nitrogen and lactose percent were determined in milk samples with MilkoScan FT 6000 Series mid-range infrared Fourier transform infrared-based spectrometers (Foss, Hillerod, Denmark) by Valacta Laboratories.

### Fatty acids analysis and quantification

The milk samples were prepared for analysis using alkali hydrolysis followed by methylation as described in Association of Analytical Communities (AOAC) Method 991.39 [33]. Briefly, 1 ml of cow milk was transferred into a 15 ml screwcap centrifuge tube in triplicates and 3 ml of 0.5N NaOH in methanol was added into each tube and mixed thoroughly by shaking for 30 s. Tubes were incubated in a water bath at 85 °C for 10 min and cooled at ambient temperature. In a fume hood, 1 ml of boron trifluoride (BF_3_) was added, mixed thoroughly by shaking and incubated in a water bath at 85 °C for 10 min. Samples were allowed to cool to room temperature followed by addition of 3 ml iso-octane and 3 ml of saturated NaCl solution, mixed by shaking vigorously followed by centrifugation at 2500 rpm for 5 min. The upper layer (iso-octane) containing the fatty acid methyl esters (FAMEs) was transferred through a funnel (cotton wool was placed in the funnel and 1 g of sodium sulfate (Na_2_SO_4_) anhydrous was added on top of the cotton wool) into a test tube with a Pasteur pipette. 2 g of anhydrous Na_2_SO_4_ was added. The FAMEs were diluted to a volume of 2 ml with hexane in a volumetric flask. 1 ml of the sample was transferred into a screw cap gas chromatography (GC) sample vial and stored at − 20 °C.

The composition of individual FAMEs was analyzed by gas chromatography mass spectrometry (GC–MS). The FAMEs in hexane (1 µl) were injected in to a 7890A GC system (Agilent Technologies, USA) coupled to a 240 ion trap mass spectrometer detector (Agilent Technologies) using the Agilent 7693A automatic liquid sampler at a split ratio of 100:1. A VF5-MS (5% phenyl methylpolysiloxane), 30 m × 0.25 mm id, 0.25 µm film capillary column was used with the injector port set at 280 °C. Helium was used as carrier gas at a flow rate of 1 ml/min. The oven temperature was programmed to rise from 50 °C to 180 °C at 4 °C/min followed by an increase to 250 °C at 3 °C/min. The ion trap mass spectrometer parameters were as follow: scan range 50–540 (m/z), ionization mode EI, filament delay time 3 min and transfer line temperature, manifold temperature and trap temperature of 250 °C, 100 °C and 150 °C, respectively.

Chromatograms and spectra representing individual FAMEs were analyzed using the automated mass spectral deconvolution and identification system software (AMDIS, US). The identification of the individual FAMES was performed by comparing each of the mass spectra with the database of NIST 11 (Gaithersburg, MD, USA) and Wiley 7N (John Wiley, NY, USA) and also by comparing the calculated Kovats linear retention indices using retention times of n-alkane series against the values obtained in the NIST webbook for the same capillary column stationery phase [34]. The quantification of individual FAMEs was performed by the peak area percentage method. The fatty acid concentrations were expressed as the ratio of each individual fatty acid to the total of all fatty acids detected in the sample. The fatty acids unsaturation indices were calculated as the ratio of *cis*-9 unsaturated to *cis*-9 unsaturated + saturated for specific fatty acid pairs and multiplied by 100 [12]. We calculated the following indices: C14 index = C14:1 *cis*-9/(C14:1 *cis*-9 + C14:0) × 100 and C18 index = C18:1 *cis*-9/(C18:1 *cis*-9 + C18:0) × 100. The total index was calculated as total index = (C14:1 *cis*-9 + C18:1 *cis*-9)/(C14:1 *cis*-9 + C14:0 + C18:1 *cis*-9 + C18:0) × 100 [22].

### Genotyping of *DGAT1* K232A and *SCD1* A293V mutations

Genomic DNA was isolated from blood samples using the phenol–chloroform method [35]. DNA quality was checked on 0.8% agarose gel and the quantity was checked using the NanoDrop ND-1000 spectrophotometer (NanoDrop Technologies, Inc., USA). The *DGAT1* K232A and *SCD1* A293V genotypes were determined by the method of polymerase chain reaction–restriction fragment length polymorphisms (PCR-RFLP) [20, 30].

The primers 5′-GCACCATCCTCTTCCTCAAG-3′ (forward) and 5′-GGAAGCGCTTTCGGATG-3′ (reverse) [30] were used to amplify a 411 bp fragment of the bovine *DGAT1* gene containing the lysine/alanine substitution (exon 8) while 5′-CCCATTCGCTCTTGTTCTGT-3 (forward) and 5′-CGTGGTCTTGCTGTGGACT-3′ (reverse) [20] were used to amplify a 400 bp fragment containing the A293V polymorphism in exon 5 of the *SCD1* gene. The PCR reactions were carried out in a 30 µl volume containing 45 ng of template DNA, 15 µl of PCR Master Mix (Bioneer, Korea) and 4.5 pmol of each primer (3 pmol/µl). The PCR conditions were as follows: an initial denaturation step at 94 °C for 3 min, 35 cycles of 94 °C for 45 s, 62 °C (*DGAT1* K232A) or 64 °C (*SCD1* A293V) for 60 s, 72 °C for 60 s, and a final extension step of 72 °C for 5 min. The PCR reactions were performed in the GeneAmp PCR System 9700 (Applied Biosystems, USA). Amplification was confirmed by running the PCR products on 1.8% agarose gel and visualized with GelDoc-It^2^ Imager (Ultra-Violet Products Ltd., UK). The PCR products were purified with the QIA quick PCR Purification Kit (Qiagen, Germany).

Five microliter of purified PCR products containing the *DGAT1* K232A were digested overnight at 37 °C with 10 U of *EaeI* restriction enzyme (New England Biolabs, Inc., USA). The digested PCR products were separated on 1.8% agarose gel stained with GelRed™ (Biotium, UK) resulting in two fragments of 203 and 208 bp (seen as a single band on gel) for AA genotype, two fragments of 203/208 bp and 411 bp for KA genotype and the undigested 411 bp for KK genotype (Additional file 1: Figure a).

Similarly, 5 µl of purified PCR products containing the *SCD1* A293V mutation were digested overnight at 37 °C with 10 U of *Nco*I restriction enzyme (New England Biolabs, Inc., USA). The digested products were separated on 1.8% agarose gel stained with GelRed™ (Biotium, UK), visualized and scanned with GelDoc-It^2^ Imager (Ultra-Violet Products Ltd., UK). The digestion patterns resulted in two fragments of 200 bp for the AA genotype, undigested 400 bp fragment for the VV genotype and 400 bp and 200 bp fragments for the AV genotypes (Additional file 1: Figure b).

### Statistical analysis

The allele frequencies and test for Hardy–Weinberg Equilibrium (HWE) were performed with GENEPOP program version 1.2 [36]. To investigate the effect of breed, *SCD1* A293V and *DGAT1* K232A genotypes on milk fatty acids and milk components, the following mixed linear model with IBM SPSS version 20 software package was used:

$${{\text{Y}}_{{\text{ijklm}}}}=\upmu +{{\text{B}}_{\text{i}}}+{\text{G}}{{\text{D}}_{\text{j}}}+{\text{G}}{{\text{S}}_{\text{k}}}+{\left( {{\text{Bi}} \times {\text{Gj}}} \right)_{{\text{ij}}}}+{\left( {{\text{Bi}} \times {\text{G}}{{\text{S}}_{\text{k}}}} \right)_{{\text{ik}}}}+{\text{G}}{{\text{R}}_{\text{l}}}{{\text{A}}_{\text{m}}}+{{\text{E}}_{{\text{ijklmn}}}}$$

where Y_ijklmn_ is the observed phenotype: fat%, protein%, lactose%, milk urea nitrogen (mg/dl), individual fatty acid%, groups of fatty acids (saturated fatty acids [SFA], monounsaturated fatty acids [MUFA], polyunsaturated fatty acids [PUFA]) and unsaturation indices (C14 index, C18 index and total index), µ is the population mean, Bi is the fixed effect of Breed, GDj is the fixed effect of *DGAT1* genotypes (KK and KA), GS_k_ is the fixed effect of *SCD1* genotypes (VV, AV); (Bi × Gj)_ij_ is the fixed interaction effect between breed and *DGAT1* genotypes, (Bi × GS_k_)_ik_ is the fixed interaction effect between breed and *SCD1* genotypes, GR is the geographical region (south and north), A_m_ is the random animal effect and E_ijklmn_ is the random residual error associated with each record. The age, lactation stage and parity number were included in the initial model and were dropped out from the final model due to the absence of significant effect. The allele substitution effect of *DGAT1* (K variant) and *SCD1* (V variant) was estimated following the method of Marchitelli et al. [37] by regressing the number of copies of *DGAT1* K allele and *SCD1* V allele against each of the milk fatty acid and milk component traits separately. The phenotypes were nested within breed to obtain breed specific estimates. The results of the different effects are presented as least squares means ± standard error. The Pearson correlation indices were calculated among the various milk and fatty acids and milk component traits using the IBM SPSS version 20 software package. The principal component analysis (PCA) of the significant milk traits (Fat, protein, C14:1 *cis*-9, C16:0, C18:2 *cis*-9, *cis*-12, C18:1 *cis*-9, C14 index, C18 index, Total index, SFA, MUFA and PUFA) between breeds was done using Minitab software version 18. Significance was declared at P < 0.05.

## Results

### Effect of breed on milk component and fatty acid traits

Least square means of milk components and fatty acid profiles across breeds are reported in Table 1. White Fulani produced milk with higher content of fat compared to Borgou (P < 0.001). On the other hand, Borgou presented higher content of milk urea nitrogen than White Fulani (P < 0.001). However, no significant differences were observed for protein and lactose contents between the two breeds (Table 1). About fifteen different fatty acids were quantified with confidence in the milks of White Fulani and Borgou breeds, namely caproic acid (C6:0), caprylic acid (C8:0), capric acid (C10:0), lauric acid (C12:0), 12-methyl tridecanoic acid (C13:0), myristoleic acid (C14:1 *cis*-9), myristic acid (C14:0), pentadecanoic acid (C15:0), palmitic acid (C16:0), margaric acid (C17:0), linoleic acid (C18:2 *cis*-9, *cis*-12), oleic acid (C18:1 *cis*-9), stearic acid (C18:0), nonadecanoic acid (C19:0) and arachidic acid (C20:0). The fatty acid profiles revealed that oleic acid (16.63%) and linoleic acid (15.84%) were the most abundant fatty acids in Borgou milk while stearic acid (17.88%) and palmitic acid (16.19%) were the most abundant fatty acids in White Fulani milk. The Borgou cows produced milk with higher contents of C8:0, C10:0, C14:0, C15:0, C17:0, C18:2 *cis*-9, *cis*-12, C18:1 *cis*-9, and C19:0 compared to White Fulani cattle breed (P < 0.05). On the other hand, White Fulani produced milk with higher contents of C18:0 and C16:0 compared to Borgou breed (P < 0.001). Moreover, Borgou had higher C18 unsaturation index, total index and higher contents of PUFA than White Fulani (P < 0.05). On the contrary White Fulani produced milk with higher (P < 0.001) contents of total SFA as compared to Borgou. No significant differences between breeds were observed for C6:0, C12:0, C13:0, C14:1 *cis*-9, C20:0 and MUFA (Table 1).

**Table 1:** Effect of breed on milk production traits and individual fatty acids composition in Borgou and White Fulani Cows

Trait	Breed				P-value
	Borgou (85)	SEM	White Fulani (96)	SEM	
Milk production traits					
Fat (%)	4.51	0.19	5.49	0.19	< **0.001**
Protein (%)	3.76	0.06	3.8	0.06	0.620
Milk urea nitrogen (mg/dl)	10.33	0.33	8.04	0.33	< **0.001**
Lactose (%)	4.45	0.04	4.48	0.04	0.586
Fatty acids and unsaturation indices (%)					
Caproic acid (C6:0)	0.29	0.04	0.23	0.03	0.268
Caprylic acid (C8:0)	0.42	0.04	0.31	0.04	**0.042**
Capric acid (C10:0)	1.15	0.08	0.93	0.07	**0.047**
Lauric acid (C12:0)	1.57	0.11	1.46	0.11	0.470
12-Methyl tridecanoic acid (C13:0)	0.37	0.04	0.34	0.04	0.735
Myristoleic acid (C14:1 *cis*-9)	1.02	0.08	0.87	0.08	0.210
Myristic acid (C14:0)	12.19	0.60	9.07	0.57	< **0.001**
Pentadecanoic acid (C15:0)	4.29	0.24	2.62	0.23	< **0.001**
Palmitic acid (C16:0)	5.27	0.95	16.19	0.89	< **0.001**
Margaric acid (C17:0)	7.34	0.36	3.67	0.34	< **0.001**
Linoleic acid (C18:2 *cis*-9, *cis*-12)	15.84	0.64	9.85	0.60	< **0.001**
Oleic acid (C18:1 *cis*-9)	16.63	0.84	14.25	0.79	**0.042**
Stearic acid (C18:0)	12.96	0.90	17.86	0.84	< **0.001**
Nonadecanoic acid (C19:0)	0.96	0.13	0.53	0.13	**0.019**
Arachidic acid (C20:0)	0.20	0.03	0.24	0.03	0.207
C14 index^a^	9.69	1.17	8.48	1.10	0.452
C18 index^b^	55.61	2.11	45.37	1.98	< **0.001**
Total index^c^	40.78	1.72	35.66	1.62	**0.031**
Fatty acid groups (%)					
SFA	58.46	1.03	68.19	0.97	< **0.001**
MUFA	21.98	1.09	19.41	1.03	0.088
PUFA	19.56	0.77	12.40	0.72	< **0.001**

Bold in the table indicates P-values lower than 0.05

*SFA* saturated fatty acid, *MUFA* monounsaturated fatty acid, *PUFA* polyunsaturated fatty acid, *SEM* standard errors of the means

^a^C14 index = C14:1 *cis*-9/(C14:1 *cis*-9 + C14:0) × 100

^b^C18 index = C18:1 *cis*-9/(C18:1 *cis*-9 + C18:0) × 100

^c^Total index = (C14:1 *cis*-9 + C18:1 *cis*-9)/(C14:1 *cis*-9 + C14:0 + C18:1 *cis*-9 + C18:0) × 100

**Fig. 2:**
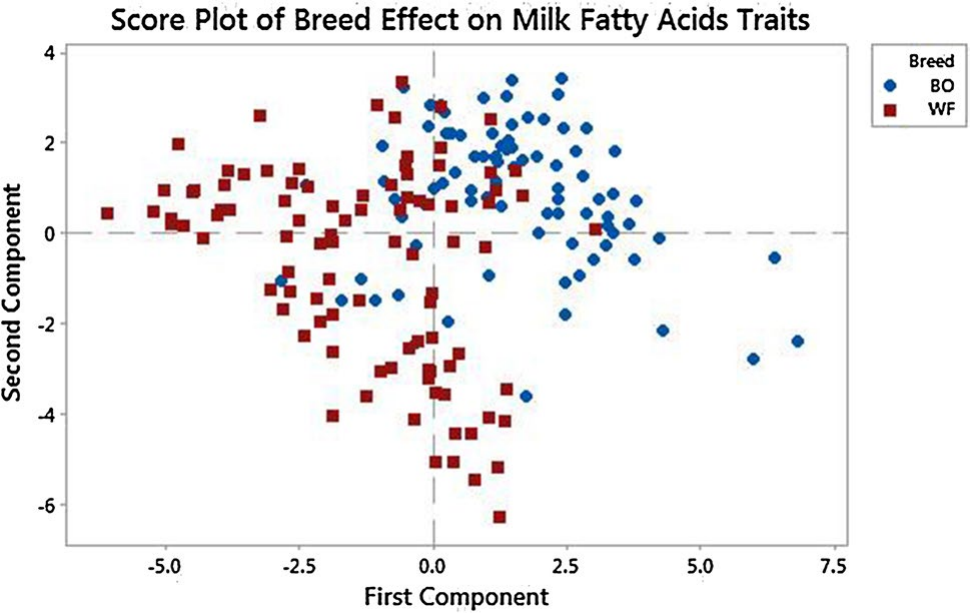
Principal component analysis of milk component and fatty acids traits in Borgou (BO) and White Fulani (WF) cows. The first component grouped most of the variables from White Fulani on the left side and Borgou on the right side

The principal component analysis (PCA) of the significant variables (Fat, protein, C14:1 *cis*-9, C16:0, C18:2 *cis*-9, *cis*-12, C18:1 *cis*-9, C14 index, C18 index, Total index, SFA, MUFA and PUFA) between breeds is presented in Fig. 2. The first component (Axis) clearly separated White Fulani from Borgou. The White Fulani population (red) was on the left side and the Borgou (blue) on the right side as shown in Fig. 2. The PCA analysis therefore showed that the two cattle populations are different for the studied milk traits.

### Effect of geographical regions

The effect of geographical regions on milk fatty acid traits is presented in Table 2. Because the White Fulani cattle are only found in the northern part of Benin, only Borgou breed was considered in the geographical effect analysis. The Borgou cows from the South of Benin showed milk with higher contents of C12:0, C18:2 *cis*-9, *cis*-12 and PUFA (P < 0.05). However, the Borgou cows from the North of Benin produced milk with higher contents of C14:1 *cis*-9, C18:1 *cis*-9, MUFA, and higher C14 index, C18 index and total index compared to Borgou cows from the South of Benin (P < 0.05).

**Table 2:** Effect of geographical regions on milk fatty acids composition traits in Borgou

Trait (%)	Geographical region				P-value
	South (32)	SEM	North (53)	SEM	
Caproic acid (C6:0)	0.23	0.05	0.32	0.04	0.117
Caprylic acid (C8:0)	0.33	0.07	0.48	0.05	0.068
Capric acid (C10:0)	1.18	0.12	1.12	0.09	0.717
Lauric acid (C12:0)	2.07	0.19	1.27	0.15	**0.002**
12-Methyl tridecanoic acid (C13:0)	0.36	0.08	0.37	0.06	0.944
Myristoleic acid (C14:1 *cis*-9)	0.70	0.12	1.21	0.09	**0.001**
Myristic acid (C14:0)	12.88	0.99	11.78	0.77	0.387
Pentadecanoic acid (C15:0)	4.00	0.49	4.46	0.38	0.454
Palmitic acid (C16:0)	5.76	1.43	4.98	1.11	0.667
Margaric acid (C17:0)	7.72	0.71	7.11	0.55	0.492
Linoleic acid (C18:2 *cis*-9–12)	17.48	1.03	14.85	0.80	**0.046**
Oleic acid (C18:1 *cis*-9)	13.69	1.13	18.40	0.88	**0.001**
Stearic acid (C18:0)	13.35	0.88	12.72	0.69	0.569
Nonadecanoic acid (C19:0)	0.79	0.27	1.06	0.21	0.436
Arachidic acid (C20:0)	0.21	0.04	0.19	0.03	0.761
C14 index^a^	5.01	2.56	12.52	1.99	**0.023**
C18 index^b^	50.83	2.38	58.50	1.85	**0.012**
Total index^c^	55.84	3.86	71.02	3.00	**0.003**
SFA	60.63	1.58	57.15	1.22	0.086
MUFA	17.87	1.43	24.46	1.11	< **0.001**
PUFA	21.50	1.22	18.39	0.95	**0.046**

Bold in the table indicates P-values lower than 0.05

*SFA* saturated fatty acid, *MUFA* monounsaturated fatty acid, *PUFA* polyunsaturated fatty acid, *SEM* standard errors of the means

^a^C14 index = C14:1 *cis*-9/(C14:1 *cis*-9 + C14:0) × 100

^b^C18 index = C18:1 *cis*-9/(C18:1 *cis*-9 + C18:0) × 100

^c^Total index = (C14:1 *cis*-9 + C18:1 *cis*-9)/(C14:1 *cis*-9 + C14:0 + C18:1 *cis*-9 + C18:0) × 100

### Effects of *SCD1* A293V polymorphism on milk traits

The frequencies of 293V were 0.84 and 0.94 in Borgou and White Fulani respectively, and the genotypes were in Hardy–Weinberg equilibrium (Table 3). Only four Borgou cows and one White Fulani cow were of AA genotype. The AA genotype was therefore not included in the association analysis. The *SCD1* VV genotype was associated with higher (P < 0.05) protein and lactose contents and lower (P < 0.05) C18:1 *cis*-9 content in White Fulani (Table 4). On the other hand, the *SCD1* AV genotype was associated with higher (P < 0.01) C14 index and total index compared to the VV genotype in Borgou (Table 4).

**Table 3:** Allele and genotype frequencies for *SCD1* A293V and *DGAT1* K232A polymorphisms in Borgou and White Fulani cows

Gene	Breed	Genotype frequencies			Allele frequencies		Chi-square^b^
		VV	AV	AA	V	A	
SCD1 A293V	Borgou (85)	61	20	4	0.84	0.16	1.78
	White Fulani (96)	85	10	1	0.94	0.06	1.19

		KK	KA	AA	K	A	
DGAT1 K232A	Borgou (83)^a^	48	31	4	0.77	0.23	0.13
	White Fulani (96)	81	14	1	0.92	0.08	0.2

Numbers in brackets correspond to sample size

^a^2 Borgou cows could not be genotyped for *DGAT1* K232 polymorphism

^b^Critical Chi square at 1 degree of freedom is 3.84

**Table 4:** Effect of *SCD1* A293V genotypes on milk components and fatty acids traits in White Fulani and Borgou cattle breeds

Trait	SCD1 genotypes in White Fulani			SCD1 genotypes in Borgou			P-value
	VV ± SE (n = 84)	AV ± SE (n = 10)	P-value	VV ± SE (n = 61)	AV ± SE (n = 20)	AA ± SE (n = 4)	
Milk production traits							
Fat (%)	4.82 ± 0.19	4.80 ± 0.53	0.887	4.74 ± 0.28	5.7 ± 0.49	3.25 ± 1.09	0.075
Protein (%)	3.90 ± 0.09	3.2 ± 0.25	**0.023**	3.84 ± 0.11	4.05 ± 0.20	3.50 ± 0.44	0.438
Lactose (%)	4.84 ± 0.04	4.60 ± 0.12	**0.024**	4.44 ± 0.12	4.65 ± 0.13	4.83 ± 0.28	0.282
Fatty acids and unsaturation indices (%)							
Caproic acid (C6:0)	0.25 ± 0.04	0.07 ± 0.12	0.366	0.25 ± 0.03	0.43 ± 0.06	0.13 ± 0.12	**0.010**
Caprylic acid (C8:0)	0.10 ± 0.03	0.00 ± 0.08	0.573	0.38 ± 0.05	0.59 ± 0.08	0.21 ± 0.18	**0.040**
Capric acid (C10:0)	0.50 ± 0.09	0.30 ± 0.27	0.671	1.14 ± 0.08	1.25 ± 0.15	0.76 ± 0.33	0.384
Lauric acid (C12:0)	0.94 ± 0.11	0.50 ± 0.32	0.300	1.68 ± 0.15	1.28 ± 0.26	1.35 ± 0.58	0.386
12-Methyl tridecanoic acid (C13:0)	0.10 ± 0.04	0.00 ± 0.10	0.646	0.38 ± 0.06	0.32 ± 0.1	0.35 ± 0.22	0.839
Myristoleic acid (C14:1 *cis*-9)	0.45 ± 0.09	0.10 ±0.25	0.365	0.95 ± 0.09	1.31 ± 0.16	0.58 ± 0.35	0.065
Myristic acid (C14:0)	4.10 ± 0.27	5.10 ± 0.80	0.469	12.54 ± 0.72	11.66 ± 1.26	9.6 ± 2.82	0.536
Pentadecanoic acid (C15:0)	2.13 ± 0.18	1.80 ± 0.53	0.682	4.13 ± 0.35	4.97 ± 0.62	3.27 ± 1.38	0.379
Palmitic acid (C16:0)	2.14 ± 0.23	1.30 ± 0.65	0.478	5.46 ± 1.01	3.05 ± 1.76	13.60 ± 3.94	0.053
Margaric acid (C17:0)	2.82 ± 0.25	3.10 ± 0.72	0.434	7.01 ± 0.51	8.56 ± 0.89	6.26 ± 1.99	0.278
Linoleic acid (C18:2 *cis*-9, *cis*-12)	3.44 ± 0.29	3.30 ± 0.83	0.650	16.09 ± 0.76	15.11 ± 1.34	15.70 ± 2.98	0.815
Oleic acid (C18:1 *cis*-9)	2.92 ± 0.27	3.70 ±0.78	**0.039**	16.08 ± 0.85	19.14 ± 1.48	12.43 ± 3.32	0.093
Stearic acid (C18:0)	2.82 ± 0.26	2.40 ± 0.74	0.821	13.32 ± 0.64	11.65 ± 1.11	13.87 ± 2.49	0.400
Nonadecanoic acid (C19:0)	0.19 ± 0.09	0.00 ± 0.79	0.753	0.88 ± 0.20	1.05 ± 0.35	1.78 ± 0.77	0.512
Arachidic acid (C20:0)	0.03 ± 0.02	0.00 ± 0.05	0.878	0.19 ± 0.03	0.16 ± 0.05	0.45 ± 0.11	0.062
C14 index^a^	8.80 ± 0.54	6.10 ± 1.57	0.269	6.93^a^ ± 1.81	18.94^b^ ± 3.15	5.57^a^ ± 7.05	**0.005**
C18 index^b^	46.59 ± 2.53	39.77 ± 7.35	0.515	54.71 ± 1.77	59.85 ± 3.09	48.22 ± 6.90	0.199
Total index^c^	36.56 ± 2.00	31.46 ± 5.80	0.598	61.64^a^ ± 2.80	78.79^b^ ± 4.89	53.79^a^ ± 10.94	**0.007**
SFA	67.96 ± 1.08	68.56 ± 3.14	0.968	47.36 ± 0.88	44.96 ± 1.54	51.61 ± 3.44	0.161
MUFA	20.02 ± 1.21	16.36 ± 3.52	0.499	17.03 ± 0.86	20.45 ± 1.50	13.02 ± 3.35	0.058
PUFA	12.02 ± 0.78	15.08 ± 2.26	0.332	16.09 ± 0.76	15.11 ± 1.34	15.70 ± 2.98	0.815

Bold in the table indicates P-values lower than 0.05

SCD1 AA genotype was not included in the analysis for While Fulani because only one individual of White Fulani breed was AA. Means with different superscript letters across genotypes differ significantly

*SFA* saturated fatty acid, *MUFA* monounsaturated fatty acid, *PUFA* polyunsaturated fatty acid, *SE* standard error of the mean

^a^C14 index = C14:1 *cis*-9/(C14:1 *cis*-9 + C14 :0) × 100

^b^C18 index = C18:1 *cis*-9/(C18:1 *cis*-9 + C18 :0) × 100

^c^Total index= (C14:1 *cis*-9 + C18:1 *cis*-9)/(C14:1 *cis*-9 + C14 :0 + C18:1 *cis*-9 + C18:0) × 100

The estimates of the *SCD1* 293V allele substitution effect in Borgou breed are presented in Table 5. In comparison to the A allele, the V allele was associated with decrease in C14 index (− 5.68%, P < 0.05). However, no significant 293V allele substitution effect was observed for C18 and total indices, and MUFA in Borgou (Table 5).

**Table 5:** Effect of *SCD1* V and *DGAT1* K alleles substitution on fatty acids unsaturation in Borgou and White Fulani cattle breeds respectively

Traits	*SCD1* V (Borgou)		P-value	*DGAT1* K (White Fulani)		P-value
	Estimates	SEM		Estimates	SEM	
C14 index	− 5.68	2.7	**0.048**	2.23	1.26	0.078
C18 index	− 0.96	0.27	0.724	− 12.16	5.84	**0.040**
Total index	− 2.11	0.24	0.385	− 12.81	4.53	**0.008**
SFA	0.14	0.01	0.916	5.41	2.4	**0.031**
MUFA	− 0.708	1.33	0.596	− 8.09	2.72	**0.004**

Bold in the table indicates P-values lower than 0.05

*SFA* saturated fatty acid, *MUFA* monounsaturated fatty acid, *SEM* standard error of the means

### Effects of the *DGAT1* K232A polymorphism

The frequencies of 232 K were 0.77 and 0.92 in Borgou and White Fulani respectively, and the genotypes were in Hardy–Weinberg equilibrium (Table 3). The *DGAT1* K232A polymorphism did not significantly affect milk composition, fatty acid profiles and unsaturation indices in Borgou breed and White Fulani (Additional file 2: Table S1). However, the P-values for C14:0, C15:0 and C19:0 tended towards significance (P < 0.1) in Borgou where the *DGAT1* KK genotype seems to show higher C14:0 and C15:0, and lower C19:0 contents (Additional file 2: Table S1). However, allele substitution effects indicated that the *DGAT1* 232K allele was associated with increased total saturated fatty acid (SFA, + 5.41%, P < 0.05), and with decreased C18 index (− 12.16%, P < 0.05), total index (− 12.81%, P < 0.01) and MUFA (− 8.09%, P < 0.01) in White Fulani breed (Table 5).

### Phenotypic correlations

The phenotypic correlations between milk component and fatty acid traits in Borgou cows are presented in Table 6. In Borgou, the fat percentage showed significantly (P < 0.05) positive correlation with protein content (0.54), C14 index (0.45), C18:1 *cis*-9 (0.24), and MUFA (0.28) and negative correlations with C16:0 (− 0.22) and PUFA (− 0.25). The C14:1 *cis*-9 showed positive and moderate correlation (P < 0.01) with C14 index (0.33) and C18 index (0.31) but a negative correlation with C16:0 (− 0.37, P < 0.001). The correlations of C16:0 were negative between all the traits except for SFA (0.48), (Table 6). The C18:2 *cis*-9, *cis*-12 was negatively correlated (P < 0.01) with C18:1 *cis*-9, SFA and MUFA. The C18:1 *cis*-9 showed high significant and positive correlation (P < 0.001) with C18 index, total unsaturation index and MUFA. On the other hand, high significant and negative correlation (P < 0.001) was observed between C18:1 *cis*-9 and SFA. The C14 index showed moderate and positive correlation (P < 0.05) with C18 index (0.27), total unsaturation index (0.52) and with MUFA (0.38). The total index showed high positive correlation (P < 0.001) with MUFA (0.88) and negative correlation (P < 0.001) with SFA (− 0.71).

**Table 6:** Phenotypic coefficient of correlations (Pearson) in Borgou

Trait	Fat (%)	Protein (%)	C14:1 *cis*-9	C16:0	C18:2 *cis*-9, *cis*-12	C18:1 *cis*-9	C14 index	C18 index	Total index	SFA	MUFA	PUFA
Fat (%)		0.54***	0.45***	− 0.22*	− 0.25*	0.24*	0.14	0.20	0.15	− 0.07	0.28*	− 0.25*
Protein (%)	0.54***		0.17	− 0.17	− 0.00	0.17	0.03	0.05	0.07	− 0.16	0.17	− 0.01
C14:1 *cis*-9	0.45***	0.17		− 0.37***	− 0.11	0.10	0.33**	0.31**	0.08	− 0.10	0.20	− 0.12
C16:0	− 0.23*	− 0.17	− 0.37***		− 0.38***	− 0.19	− 0.11	− 0.15	− 0.01	0.48***	− 0.19	− 0.38***
C18:2 *cis*-9, *cis*-12	− 0.25*	− 0.00	− 0.11	− 0.38**		− 0.31**	− 0.03	− 0.13	− 0.17	− 0.44***	− 0.35**	1.00***
C18:1 *cis*-9	0.24*	0.17	0.10	− 0.19	− 0.31**		0.37***	0.75**	0.90***	− 0.72***	0.99***	− 0.31**
C14 index	0.14	0.03	0.33**	− 0.11	− 0.03	0.37**		0.27*	0.52***	− 0.34**	0.38***	− 0.04
C18 index	0.20	0.05	0.31**	− 0.15	− 0.13	0.75**	0.271*		0.83***	− 0.63***	0.76***	− 0.13
Total index	0.15	0.07	0.08	− 0.01	− 0.17	0.90**	0.52***	0.83***		− 0.71***	0.88***	− 0.17
SFA	− 0.07	− 0.16	− 0.10	0.48***	− 0.44***	− 0.72***	− 0.34**	− 0.63***	− 0.71***		− 0.69***	− 0.44***
MUFA	0.28*	0.17	0.2	− 0.19	− 0.35**	0.99***	0.38***	0.76***	0.88***	− 0.69***		− 0.35**
PUFA	− 0.25*	− 0.01	− 0.12	− 0.38***	1.00***	− 0.31**	− 0.04	− 0.13	− 0.17	− 0.44***	− 0.35**	

*SFA* saturated fatty acid, *MUFA* monounsaturated fatty acid, *PUFA* polyunsaturated fatty acid

*P < 0.05; **P < 0.01; ***P < 0.001

Table 7 presents the phenotypic correlations between milk component and fatty acid traits in White Fulani cows. Fat content showed high significant and positive correlation (P < 0.01) with protein content, C14:1 *cis*-9 and C14 index (Table 7). The protein percentage was moderately and positively correlated (P < 0.05) with C14:1 *cis*-9 and C14 index. The C18:2 *cis*-9-12 was negatively correlated (P < 0.05) with C18 index (− 0.24) and PUFA (− 0.42) while positively correlated (P < 0.05) to C14 index (0.24) and SFA (0.31). The total index showed high positive correlation (P < 0.001) with MUFA (0.94), negative correlation (P < 0.0.01) with SFA (**−** 0.82) and PUFA (**−** 0.34) (Table 7).

**Table 7:** Phenotypic coefficient of correlations (Pearson) in White Fulani

Trait	Fat (%)	Protein (%)	C14:1 *cis*-9	C16:0	C18:2 *cis*-9, *cis*-12	C18:1 *cis*-9	C14 index	C18 index	Total index	SFA	MUFA	PUFA
Fat (%)		0.62***	0.40***	0.08	0.19	0.00	0.29**	− 0.06	− 0.13	0.04	− 0.11	0.12
Protein (%)	0.62***		0.34**	0.09	0.13	− 0.13	0.26*	− 0.05	− 0.08	0.07	− 0.03	− 0.05
C14:1 *cis*-9	0.40***	0.34**		− 0.05	− 0.07	0.00	0.43***	0.11	− 0.10	− 0.09	− 0.10	0.27**
C16:0	0.08	0.09	− 0.05		0.12	0.14	0.04	− 0.20*	− 0.16	0.09	− 0.19	0.18
C18:2 *cis*-9, *cis*-12	0.19	0.13	− 0.07	0.12		− 0.12	0.24*	− 0.24*	− 0.10	0.31**	− 0.01	− 0.42***
C18:1 *cis*-9	0.00	− 0.13	0.00	0.14	− 0.12		0.04	− 0.162	− 0.28**	0.18	− 0.40***	0.37***
C14 index	0.29**	0.26*	0.43**	0.03	0.24*	0.04		− 0.17	− 0.17	0.13	− 0.16	0.08
C18 index	− 0.06	− 0.05	0.11	− 0.20	− 0.24	− 0.16	− 0.17		0.93**	− 0.83***	0.82***	− 0.14
Total index	− 0.13	− 0.07	− 0.10	− 0.16	− 0.10	− 0.28**	− 0.17	0.93***		− 0.82**	0.94***	− 0.34**
SFA	0.04	0.07	− 0.09	0.09	0.31**	0.18	0.13	− 0.83***	− 0.82**		− 0.78***	− 0.18
MUFA	− 0.11	− 0.03	− 0.1	− 0.19	− 0.01	− 0.40***	− 0.16	0.82***	0.94***	− 0.78***		− 0.49***
PUFA	0.12	− 0.05	0.27**	0.18	− 0.42***	0.37***	0.08	− 0.14	− 0.34**	− 0.18	− 0.49***	

*SFA* saturated fatty acid, *MUFA* monounsaturated fatty acid, *PUFA* = polyunsaturated fatty acid

*P < 0.05; ^**^P < 0.01; ^***^P < 0.001

## Discussion

### Effect of breed and geographical regions

In the present study, we analyzed the milk and fatty acid composition as well as fatty acid unsaturation indices in indigenous White Fulani and Borgou cows in Benin. White Fulani produced milk with high (P < 0.001) fat content than Borgou. Significant differences between breeds were observed for individual fatty acids, fatty acid unsaturation indices and fatty acid groups. The milk of Borgou breed had higher MUFA and PUFA content and C18 and total indices than White Fulani. Breed effect on fatty acid composition was reported in the meat of Borgou and White Fulani cattle in Benin [38], in the milk of South African indigenous cattle breeds [39] and Italian Holstein–Friesian, Brown Swiss, Simmental and Alpine cattle breeds [40]. In this study, White Fulani presented the highest content of SFA (68.19 vs. 58.46%, P < 0.001) and the lowest content of PUFA (12.40 vs. 19.56%, P < 0.001) compared to Borgou, and similar MUFA content (19.41 vs. 21.98%, P > 0.05) for both breeds. These results corroborate previous studies in Benin indicating that the meat of White Fulani bulls had higher SFA content (49.68%) compared to Borgou (43.03%) and similar MUFA content (33.60% vs. 33.43%) [38]. In the current study, Borgou presented higher C18 index (P < 0.001) and total unsaturation index (P < 0.05) than White Fulani. The unsaturation or desaturation index of a specific fatty acid represents the ratio of the concentration of the monounsaturated product to the sum of the monounsaturated and the saturated substrate [22]. Considering human health aspects, increasing the amount of unsaturated fatty acids as well as unsaturation indices is an important selection objective [10]. Borgou presented lower SFA (58.46%) than the values of 64%, 63.7%; 60.9% and 71.9% reported in milk of free-ranging South African indigenous Boran, Nguni, Tuli and Afrikaner cattle breeds, respectively [39]. Moreover, Borgou and White Fulani presented lower MUFA content (21.98% and 19.41%, respectively) than South African indigenous cattle (MUFA content ranged from 25.7% in Afrikaner to 36.5% in Tuli breed) [39]. On the contrary, Borgou and White Fulani produced milk with C18:2 *cis*-9, *cis*-12 of 15.84% and 9.85% respectively, much higher than the range of 1.3 to 1.7% observed in South African indigenous Boran, Nguni, Tuli and Afrikaner [39]. The difference in C18:2 *cis*-9, *cis*-12 content between Borgou/White Fulani and South African indigenous cattle may be due to the lower sample size in their study being 6 Boran, 9 Nguni, 10 Tuli and 6 Afrikaner [39]. Several factors influence milk fatty acid composition such as species, breed, individual variability, nutrition, stage of lactation, parity and season [40, 41]. Studied animals were raised in the traditional system on natural grazing without concentrate supplementation and they were sampled at the same period eliminating the effect of season. The observed differences between breeds would therefore be due to their genetic background. However, the differences in fatty acid composition between the two breeds could also be due to the fatty acid composition of the forage consumed by the cows on natural grazing. The White Fulani and Borgou are raised in different agro-ecological zones with different floristic composition. The White Fulani cattle are found in the Northern part of Benin while Borgou cattle are found throughout the country. The forage species and variety, climate and stage of growth are important factors that affect fatty acid content and composition of forage [38, 42] and can therefore affect the milk fatty acid composition of the cows. The Borgou cows from the North of Benin produced milk with higher C14:1 *cis*-9, C18:1 *cis*-9, C14 unsaturation index, C18 unsaturation index, total unsaturation index and MUFA contents than Borgou cows from the South of Benin. Consequently, milk from Borgou cows raised in the North of Benin seems to be healthier than from Borgou cows raised in the South of Benin due to its higher content of MUFA and higher unsaturation indices.

The suggested favorable combination of bovine milk fatty acid composition for human health enhancement is ~ 30% SFA, 60% MUFA, and 10% PUFA [43]. In the present study, the milk fatty acid compositions were 58.48% SFA, 21.98% MUFA and 19.56 PUFA for Borgou and 68.19% SFA, 19.41% MUFA and 12.40% PUFA for White Fulani. It’s clear that the current milk fatty acid composition of Borgou and White Fulani is far from optimal and there is need for modification towards an ideal profile. Linoleic acid (a PUFA) and oleic acid (a MUFA) have been associated with decreased serum total cholesterol and low-density lipoprotein cholesterol levels and reduced risk of coronary heart diseases in humans [44]. Oleic acid has anticancer and antiatherogenic properties [45]. Linoleic acid, an essential fatty acid in the omega-6 family is associated with reduced incidence of type 2 diabetes through its ability to improve sensibility to insulin [46]. On the other hand, palmitic acid (C16:0), considered as hypercholesterolemic is responsible for the increase in the concentration of low density lipoproteins (LDL) that are associated with coronary heart diseases in humans [47]. Borgou milk with its higher linoleic acid, oleic acid and lower total SFA contents as compared to White Fulani with higher total SFA and palmitic acid contents, may be preferred by consumers than White Fulani milk.

### Effects of *SCD1* A293V and *DGAT1* K232A polymorphisms

The frequencies of *SCD1* 293V were 0.84 and 0.94 in Borgou and White Fulani, respectively. A higher frequency of the V allele (0.82) is also reported in Italian Brown cows [15] while a higher frequency of the A allele has been reported in Dutch Holstein–Friesian heifers (0.73), Italian Holsteins (0.57), and Canadian Jersey cows (0.80) [10, 20, 22]. The difference in *SCD1* A293V allele frequencies between the studied indigenous breeds and western breeds can be explained by a breed specific effect. The *SCD1* AV genotype was associated with higher C14 and total unsaturation index compared to the VV genotype in Borgou breed. This result did not agree with Conte et al. [15] who associated *SCD1* VV genotype with higher C14 index in Italian Brown cows. Moreover, Kgwatalala et al. [20] showed that the AA genotype of *SCD1* was associated with higher C14 index. The effect of *SCD1* A293V genotypes on C14 index seems to vary from one breed to another. In the current study, *SCD1* genotypes did not significantly affect protein or fat percentage in Borgou (Table 4) which is similar to the results of Schennink et al. [10] in Dutch Holstein–Friesian heifers. However, in the present study, the *SCD1* V allele had significant negative association (− 5.68%, P < 0.05) with C14 index compared to the A allele in Borgou. The allele A of *SCD1* is therefore significantly associated with 5.68% more C14 index in Borgou. The positive significant association between the allele A of *SCD1* and C14:1 *cis*-9 and C14 index has been reported previously [10, 20, 22]. However, allele A of *SCD1* did not show significant effect on C14:1 *cis*-9 in this study. This may be explained by the large sample sizes used in the other studies, namely 1725 Dutch Holstein–Friesian heifers [10], 297 Italian Holstein Friesian cows [22] and 525 Canadian Jersey cows [20]. Also, significant associations of the *SCD1* A293V polymorphism with C10 index, C12 index, C16 index and C18 index has been reported [10, 20]. However, no significant effect of *SCD1* polymorphism was observed for C18 index in this study.

The frequencies of *DGAT1* 232K were 0.77 and 0.92 in Borgou and White Fulani breeds respectively. A higher frequency of the K allele in Borgou and White Fulani breeds in Benin [32] and in Sudanese Butana and Kenana cattle breeds have been reported previously [31]. However, a lower frequency of *DGAT1* K allele (0.40) was reported in Dutch Holstein–Friesian heifers [10]. In this study, the *DGAT1* K allele was associated with lower C18 index (P < 0.05), total unsaturation index (P < 0.01), and MUFA (P < 0.01), and with higher SFA (P < 0.05) in White Fulani breed. These results are similar to a reported by Schennink et al. [21], who studied 1762 Dutch Holstein Friesian cows and found that the *DGAT1* 232K allele was associated with more saturated fatty acid. However, no significant effect of *DGAT1* 232K allele on C18 and total unsaturation indices was found by Schennink et al. [21]. Similar to our data, Schennink et al. [10] showed that the *DGAT1* K allele was associated with lower C18 and total unsaturation indices. The majority of milk fatty acids are present in the form of triacylglycerols and the DGAT1 enzyme plays an important role during the last step of triglyceride synthesis. The *DGAT1* K232A polymorphism was reported to have significant association with milk fatty acid composition and unsaturation indices [15]. However, we did not observed significant association of *DGAT1* K232A polymorphism with individual fatty acids but significant associations with SFA and MUFA was observed. This is conceivable because the effect of DGAT1 on fatty acid composition and saturation may be due to a higher activity and alteration of specificity of DGAT1 enzyme [21] which may vary between breeds. The discussion of our results on *DGAT1* K232A polymorphism and fatty acid traits was limited to western dairy breeds because of the scarcity of data on African indigenous cattle breeds.

### Phenotypic correlations

The Pearson correlation coefficient between total SFA and total PUFA was negative and moderate (**−** 0.44) in Borgou. Similar correlation (− 0.34) has been observed between total SFAs and total PUFAs in Canadian Holsteins [48]. The fat percentage showed positive correlations with C14:1 *cis*-9 (0.45) and C18:2 *cis*-9, *cis*-12 (0.24) and negative correlation with total PUFAs (− 0.25) in Borgou. Accordingly, an increase in the fat content of Borgou milk will lead to slightly higher C14:1 *cis*-9 and C18:2 *cis*-9, *cis*-12 contents and decreased total PUFA content. The fat content positively affect the price of milk in developed countries, therefore increasing PUFAs (decreasing fat content) should have negative economic impact [49]. However, in the African context, and in Benin in particular, the price of milk is not influenced by its fat content and hence decreasing total fat content for increased PUFAs in Borgou milk will be beneficial for human health and will not negatively affect farm incomes. On the contrary, increasing fat and protein percentage in White Fulani breed will lead to slightly higher increase in C14:1 *cis*-9 and C14 index due to the moderate positive correlation observed between the traits (Table 6). In the present study, C16:0, showed significant and negative correlations with fat percentage (− 0.23), C14:1 *cis*-9 (− 0.37), C18:2 *cis*-9, *cis*-12 (− 0.38) and positive correlation with total SFA (0.48) in Borgou breed. This implies that decreasing C16:0 will lead to increase in fat percentage, C14:1 *cis*-9, C18:2 *cis*-9, *cis*-12 and decrease in total SFAs in Borgou which would be an important selection goal.

## Conclusion

This study has revealed significant differences in milk components, milk fatty acid composition and unsaturation indices between White Fulani and Borgou indigenous cattle breeds in Benin. The Borgou milk contained higher linoleic acid, higher oleic acid and lower total SFA compared to White Fulani which are beneficial traits for human health. The *SCD1* AV genotype was associated with higher C14 and total indices; and the *SCD1* V allele was associated with decrease in C14 index in Borgou. In White Fulani breed, the *SCD1* VV genotype was associated with lower C18:1 *cis*-9 content while the *DGAT1 K* allele was associated with increased total SFA, and decreased C18 unsaturation index, total unsaturation index and total MUFA. *SCD1* A293V and *DGAT1* K232A polymorphisms may serve as potential genetic markers in a breeding program to improve milk fatty acids traits in indigenous cattle breeds in Benin. However, further studies with a large population of the Borgou and White Fulani breeds are needed to better understand the genetic variability of their milk fatty acids and association with genetic polymorphisms in *SCD1* and *DGAT1*genes.

## Electronic supplementary material

Below is the link to the electronic supplementary material.

**Additional file 1: Figure (a)** *DGAT1* K232A polymorphisms: agarose gel separation showing a 411 bp fragment representing KK genotype, 203/208 fragments representing AA genotype and 203/208 bp and 411 bp fragments representing KA genotype, the uncut 411-bp fragment represents the Lysine variant (232K), whereas digested fragments represent the Alanine variant and **(b)** *SCD1* A293V polymorphisms: agarose gel separation showing a 200 bp fragment representing the AA genotype, undigested 400 bp fragment representing the VV genotype and 400 bp and 200 bp fragments representing the AV genotype. The uncut 400-bp fragment represents the Valine variant (293V), whereas digested fragments represent the Alanine variant. (JPG 15 KB)

**Additional file 2: Table S1**. Effect of *DGAT1* K232A genotypes on milk components and fatty acids traits in White Fulani and Borgou cattle breeds (DOCX 25 KB)

## Abbreviations

AMDIS: Automated mass spectral deconvolution and identification system
AOAC: Association of analytical communities
DGAT1: Acyl CoA: diacylglycerol acyltransferase 1
GC-MS: Gas chromatography–mass spectrometry
HWE: Hardy–Weinberg Equilibrium
MUFA: Monounsaturated fatty acid
NIST: National Institute of Standards and Technology
PCA: Principal components analysis
PCR: Polymerase chain-reaction
PUFA: Polyunsaturated fatty acid
RFLP: Restriction fragment length polymorphism
SCD1: Stearoyl-CoA desaturase 1
SFA: Saturated fatty acid
