# Supplementary figures and images for: Milk fatty acid variability and association with polymorphisms in SCD1 and DGAT1 genes in White Fulani and Borgou cattle breeds
Source: Mol Biol Rep. 2018 Aug 30;45(6):1849–62. doi: 10.1007/s11033-018-4331-4 (PMC6267235; doi:10.1007/s11033-018-4331-4)

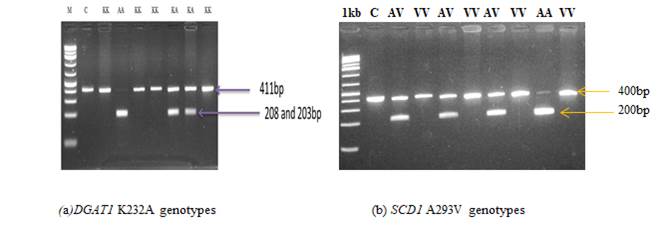

Supplement: Supplementary file 1 — Additional file 1: Figure (a) DGAT1 K232A polymorphisms: agarose gel separation showing a 411 bp fragment representing KK genotype, 203/208 fragments representing AA genotype and 203/208 bp and 411 bp fragments representing KA genotype, the uncut 411-bp fragment represents the Lysine variant (232K), whereas digested fragments represent the Alanine variant and (b) SCD1 A293V polymorphisms: agarose gel separation showing a 200 bp fragment representing the AA genotype, undigested 400 bp fragment representing the VV genotype and 400 bp and 200 bp fragments representing the AV genotype. The uncut 400-bp fragment represents the Valine variant (293V), whereas digested fragments represent the Alanine variant. (JPG 15 KB) [file 11033_2018_4331_MOESM1_ESM.jpg]
