# Supplementary material for: Milk fatty acid variability and association with polymorphisms in SCD1 and DGAT1 genes in White Fulani and Borgou cattle breeds
Source: Mol Biol Rep. 2018 Aug 30;45(6):1849–62. doi: 10.1007/s11033-018-4331-4 (PMC6267235; doi:10.1007/s11033-018-4331-4)
Supplement: Supplementary file 2 — Additional file 2: Table S1. Effect of DGAT1 K232A genotypes on milk components and fatty acids traits in White Fulani and Borgou cattle breeds (DOCX 25 KB) [file 11033_2018_4331_MOESM2_ESM.docx]

**Title:** Milk fatty acid variability and association with polymorphisms in *DGAT1* and *SCD1* genes in White Fulani and Borgou cattle breeds

**Journal name:** Molecular Biology Reports

**Isidore Houaga^1, 2*^, Anne W.T. Muigai^3^,** [**Fredrick M. Ng'ang'a**](https://www.google.com/url?sa=t&rct=j&q=&esrc=s&source=web&cd=1&cad=rja&uact=8&ved=0ahUKEwiQtc-1sLzYAhUBbxQKHVSFDXgQFggpMAA&url=http%3A%2F%2Fhub.africabiosciences.org%2Faboutbeca%2Fbecahub-staff%2F98%3Fview%3Dperson&usg=AOvVaw1Q_pHrvZASzYf51Hl3TelU)**^4^, Eveline M. Ibeagha-Awemu^5^, Martina Kyallo^4^, Issaka A.K. Youssao^2^ and Francesca Stomeo^4^**

**Corresponding author:**

Isidore HOUAGA

^1^Department of Molecular Biology and Biotechnology, Pan African University Institute of Basic Sciences, Technology and Innovation, PAUSTI-JKUAT, P.O. Box 62000-200 Nairobi, Kenya

E-mail: [houaga.isidore@students.jkuat.ac.ke](mailto:houaga.isidore@students.jkuat.ac.ke) / [ihouaga@gmail.com](mailto:ihouaga@gmail.com)

**Table S1** Effect of *DGAT1* K232A genotypes on milk components and fatty acids traits in White Fulani and Borgou cattle breeds

| **Trait** | ***DGAT1* genotypes in White Fulani** | | | ***DGAT1* genotypes in Borgou** | | |  |
| --- | --- | --- | --- | --- | --- | --- | --- |
|  | KK ± SE  (n = 80) | KA ± SE  (n = 12) | p-value | KK ± SE  (n = 48) | KA ± SE  (n = 31) | AA ± SE  (n = 4) | p-value |
| **Milk production traits** |  |  |  |  |  |  |  |
| Fat (%) | 4.88 ± 0.19 | 4.58 ± 0.48 | 0.467 | 4.85 ± 0.32 | 4.84 ± 0.40 | 5.5 ± 1.17 | 0.850 |
| Protein (%) | 3.85 ± 0.09 | 3.67 ± 0.24 | 0.471 | 3.94 ± 0.13 | 3.81 ± 0.16 | 4.00 ± 0.44 | 0.786 |
| Lactose (%) | 4.81 ± 0.05 | 4.75 ± 0.12 | 0.783 | 4.69 ± 0.19 | 4.57 ± 0.15 | 4.67 ± 0.31 | 0.870 |
| **Fatty acids and unsaturation indices (%)** |  |  |  |  |  |  |  |
| Caproic acid (C6:0) | 0.26 ± 0.04 | 0.10 ± 0.11 | 0.385 | 0.32 ± 0.04 | 0.22 ± 0.05 | 0.28 ± 0.13 | 0.229 |
| Caprylic acid (C8:0) | 0.34 ± 0.04 | 0.18 ± 0.11 | 0.378 | 0.48 ± 0.05 | 0.35 ± 0.07 | 0.23 ± 0.19 | 0.181 |
| capric acid (C10:0) | 0.99 ± 0.09 | 0.71± 0.23 | 0.465 | 1.21 ± 0.10 | 1.02 ± 0.12 | 1.14 ± 0.33 | 0.476 |
| lauric acid (C12:0) | 1.51 ± 0.11 | 1.28 ± 0.28 | 0.692 | 1.38 ± 0.16 | 1.83 ± 0.20 | 1.94 ± 0.57 | 0.197 |
| 12-Methyl Tridecanoic acid (C13:0) | 0.36 ± 0.04 | 0.35 ± 0.10 | 0.916 | 0.37 ± 0.06 | 0.34 ± 0.08 | 0.66 ± 0.22 | 0.397 |
| Myristoleic acid (C14:1 *cis*-9) | 0.95 ± 0.09 | 0.6 ± 0.23 | 0.271 | 1.13 ± 0.10 | 0.84 ± 0.13 | 1.07 ± 0.36 | 0.204 |
| Myristic acid (C14:0) | 9.42 ± 0.63 | 8.09 ± 1.62 | 0.637 | 13.33 ± 0.80 | 10.44 ± 1.00 | 12.29 ± 2.79 | **0.086** |
| pentadecanoic acid (C15:0) | 2.69 ± 0.19 | 2.57 ± 0.49 | 0.774 | 4.85 ± 0.39 | 3.75 ± 0.49 | 2.57 ± 1.36 | **0.097** |
| Palmitic acid (C16:0) | 16.06 ± 1.06 | 15. 79 ± 2.73 | 0.950 | 4.77 ± 1.18 | 6.48 ± 1.47 | 4.54 ± 4.09 | 0.649 |
| Margaric acid (C17:0) | 3.69 ± 0.29 | 4.10 ± 0.76 | 0.788 | 6.89 ± 0.57 | 7.82 ± 0.71 | 7.17 ± 1.99 | 0.599 |
| Linoleic acid (C18:2 *cis*-9, *cis*-12) | 10.24 ± 0.66 | 9.02 ± 1.70 | 0.500 | 15.80 ± 0.87 | 15.65 ± 1.08 | 17.54 ± 3.00 | 0.839 |
| Oleic acid (C18:1 *cis*-9) | 13.10 ± 0.92 | 18.52 ± 2.38 | **0.065** | 16.76 ± 0.99 | 16.46 ± 1.23 | 16.26 ± 3.44 | 0.976 |
| Stearic acid (C18:0) | 18.16 ± 1.16 | 16.07 ± 2.99 | 0.808 | 12.37 ± 0.72 | 13.85 ± 0.90 | 12.49 ± 2.51 | 0.433 |
| Nonadecanoic acid (C19:0) | 0.53 ± 0.10 | 0.59 ± 0.25 | 0.973 | 0.74 ± 0.21 | 0.99 ± 0.26 | 2.44 ± 0.72 | **0.076** |
| Arachidic acid (C20:0) | 0.25 ± 0.03 | 0.23 ± 0.08 | 0.836 | 0.18 ± 0.03 | 0.22 ± 0.04 | 0.25 ± 0.12 | 0.625 |
| C14 index^1^ | 8.97 ± 0.55 | 7.13 ± 1.41 | 0.372 | 9.66 ± 2.20 | 10.23 ± 2.73 | 7.60 ± 7.61 | 0.946 |
| C18 index^2^ | 43.33 ± 2.56 | 53.75 ± 6.60 | 0.304 | 57.23 ± 2.04 | 53.46 ± 2.54 | 53.81 ± 7.06 | 0.497 |
| Total index^3^ | 33.56 ± 1.97 | 43.64 ± 5.10 | 0.138 | 40.58 ± 1.84 | 41.25 ± 2.29 | 40.07 ± 6.37 | 0.967 |
| SFA^4^ | 54.25 ± 0.84 | 50.04 ± 2.17 | 0.170 | 58.26 ± 1.33 | 59.02 ± 1.66 | 56.86 ± 4.61 | 0.880 |
| MUFA^5^ | 14.05 ± 0.91 | 19.12 ± 2.35 | **0.085** | 22.26 ± 1.27 | 21.58 ± 1.59 | 21.72 ± 4.41 | 0.945 |
| PUFA^6^ | 10.24 ± 0.66 | 9.02 ± 1.70 | 0.500 | 19.48 ± 1.03 | 19.40 ± 1.28 | 21.43 ± 3.56 | 0.862 |

^1^ C14 index=C14:1 *cis*-9/ (C14:1 *cis*-9+ C14 :0) × 100. ^2^ C18 index= C18:1 *cis*-9/ (C18:1 *cis*-9+ C18 :0) × 100. ^3^ Total index= (C14:1 *cis*-9+ C18:1 *cis*-9)/ (C14:1 *cis*-9+ C14 :0+ C18:1 *cis*-9+ C18 :0) × 100. ^5^ SFA= saturated fatty acid; ^6^MUFA= monounsaturated fatty acid; ^6^PUFA= polyunsaturated fatty acid. SE = standard error of the mean. *DGAT1* AA genotype was not included in the analysis for While Fulani because only one individual of White Fulani breed was AA.
